# Tree Age-Related Differences in Chilling Resistance and Bark-Bleeding Physiological Responses to Chemical Component and Fiber Morphology Changes in Cell Walls of *Hevea brasiliensis* Bark

**DOI:** 10.3390/plants14162531

**Published:** 2025-08-14

**Authors:** Linlin Cheng, Huichuan Jiang, Guishui Xie, Jikun Wang, Wentao Peng, Lijun Zhou, Wanting Liu, Dingquan Wu, Feng An

**Affiliations:** 1 Rubber Research Institute, Chinese Academy of Tropical Agricultural Sciences, Haikou 571101, China; 2 Hainan Danzhou Agro-Ecosystem National Observation and Research Station, Danzhou 571737, China

**Keywords:** cold damage, LT_50_, relative water content, bark photosynthetic pigment, fiber dimension, bark tensile property, correlation analysis

## Abstract

The purpose of this study was to establish the relationship between the chilling resistance of rubber trees and the bark-bleeding characteristics caused by chilling stress, considering physiological indicators in rubber tree bark, cell wall chemical components, fiber morphologies, and tensile properties. This offered a unique perspective for examining the underlying mechanisms of latex bleeding and chilling stress in *Hevea brasiliensis*. One-year-old seedlings and two-year-old twig segments in five- and twenty-one-year-old rubber trees (5YB and 21YB) were used to compare the age-mediation differences in their various parameters. Meanwhile, the LT_50_ values were calculated with Logistic regression analysis of relative electrical conductivity (REC) data under gradient low temperatures. Subsequently, changes in corresponding parameters of 1-year-old seedling stem bark at different ages were determined, and the bark-bleeding characteristics of seedlings and twig segments were analyzed under artificially simulated chilling stress, respectively. A correlation analysis between semi-lethal temperature (LT_50_) values, relative water content (RWC) values, bark-bleeding characteristics, cell-wall chemical component contents, fiber dimensions, and tensile property parameters was implemented to estimate interrelationships among them. The LT_50_ values ranged from −2.0387 °C to −0.8695 °C. The results showed that the chilling resistance order of rubber trees at different ages was as follows: 21YB (2-year-old twig bark from 21-year-old rubber trees) > 5YB (2-year-old twig bark from 5-year-old rubber trees) > SLB (semi-lignification bark in 1-year-old seedlings) > GB (green bark in 1-year-old seedlings). The chilling resistance of seedlings and twig segments in rubber trees was highly positively (*p* < 0.001) related to fiber morphologies. Chilling-induced bark-bleeding characteristics were significantly correlated (*p* < 0.001) with fiber morphologies, bark tensile properties, and cell-wall components. The analysis data in this study contribute towards building a comprehensive understanding of the mechanisms of chilling-induced bark bleeding needed not only in rubber tree cultivation but also in sustainable rubber production.

## 1. Introduction

The rubber tree (*Hevea brasiliensis*) belongs to the Euphorbiaceae family, is monoecious, undergoes cross-pollination, and possesses a chromosome count of 2*n* = 2x = 36. The rubber tree is an erect perennial tree species and native to tropical regions of South America, especially the Amazon River Basin and adjoining areas. Due to their significant economic value as the primary source of natural rubber, rubber trees were cultivated in Southeast Asian non-traditional rubber planting areas. Extensive rubber tree plantations were established between the latitudes 18 °N and 24 °N in southern China at the beginning of the 1950s. However, rubber tree plantations in Southern China are on the fringe of tropical regions, and cold waves frequently occur [1]. When exposed to temperatures below 5 °C, rubber trees will display cold damage symptoms, such as leaf and shoot dieback, necrosis of inner and outer bark, and bark bleeding throughout the tree bodies. Latex is a complex cytoplasm, which is synthesized in laticifers in rubber trees. The laticifers form an anastomosed continuous network structure. When rubber trees are exposed to chilling temperatures and subsequent rebound temperatures, the phloem and xylem have different rates of thermal expansion and contraction. The force of expansion causes the laticifers to rupture [1]. The trade-off between the bark tension and the expansive force of latex and colloids determines whether the symptom of bark bleeding can appear. If the expansive force of the latex and colloids exceeds the tension that the bark can withstand, the bark bursts, enabling the latex to exude through fissures. Consequently, the tensile property of the bark plays a prominent role in bark bleeding in rubber trees under chilling stress.

The bark bleeding on rubber tree trunks caused by cold damage prolongs the time for trees to resume latex production, and may lead to tree death in severe cases [2]. Secondly, the bark bleeding caused by low-temperature stress induces secondary pest and disease damage to rubber trees [3,4]. Thirdly, the subsequent treatment of cold-damaged trees may cause secondary mechanical injury to the tree body [1]. These factors synthetically lead to a decrease in the resistance of rubber trees, resulting in an escalation of economic loss. For this reason, breeding programs and cultivation measures are interested in bark-bleeding mechanisms under chilling stress.

Long-lived woody perennials usually have a prolonged juvenile period and are likely to have biochemistry and adaptability to withstand a wide range of biotic and abiotic stresses. Certain studies indicate that seedlings of various woody perennial species generally exhibit lower cold tolerance compared to their physiologically mature counterparts. In older *Rhododendron* plants, increased leaf cold resistance may be partly attributed to the translocation of metabolites associated with secondary cell wall and lignin formation [5]. There are trade-offs between cold resistance and the ability to accumulate biomass [6]. An optimal strategy for younger plants might be to develop as quickly as possible to escape the hard seedling phase. Contrarily, older individuals may demonstrate superior cold resistance to juvenile and younger plants [7]. Many studies have examined the relationship between tree age and cold damage. Some of them have revealed that larger and older trees may have more structural rigidity and hence stronger tolerance to cold damage [8].

The integrity and structure of the cell wall are dynamically remolded within plant development and are capable of being adjusted to various environmental stresses [9]. The primary cell walls contain a matrix of cellulose microfibrils, interspersed with hemicellulose and embedded pectin polysaccharides. As the plant cell ages, secondary walls are formed inside the primary walls, and cellulose microfibrils become embedded in lignin. Older plant tissues have higher tensile strength due to their higher levels of lignification compared with those in young tissue. To grow and develop, plant cells must enable their walls to preserve mechanical integrity and withstand internal turgor pressure. This pressure expands the restraining walls, generating immense tensile stress within the structure of the walls [10]. Under cold stress, plant cell walls change their components and properties, including increases in cell wall thickness and tensile strength and alterations in the amount of wall polysaccharides [11,12]. Mechanical properties of plant tissues and organs are prominently dependent on intrinsic structure, cell–cell adhesion, and the mechanical properties of their cell walls [13,14]. Plant cell walls perfectly combine tensile strength and extensibility, and cellulose microfibrils are their main load-bearing elements. Depending on the plant species and varieties, the structure and composition of fibers vary, and these intrinsic differences affect their homogeneity and mechanical properties. Simultaneously, these differences are significantly correlated with the functions of plant fibers [15]. Until now, the morphology of plant fibers has been correlated with their mechanical properties via data analysis.

The photosynthetic activity of stem, twig, and bark tissues is conspicuous and has been reported by several authors [16]. As the literature has described, the prominent presence of chlorophyll-containing living bark tissues, also known as chlorenchyma, facilitates the photosynthetic activity of the bark [17]. Studies have shown that bark chlorophyll content is obviously affected by the age of the stem organ [18]. Low temperatures depress the metabolic processes in plants, impeding the biosynthesis of photosynthetic pigments or boosting the degradation of pigments. The operation of some functions in bark likely brings about resource allocation tradeoffs: photosynthetic activity may necessitate a greater water content within the bark; water-storage bark is characterized by thinner, less lignified walls; and increasing bark thickness markedly boosts both the stem’s stiffness and its capacity for water storage [19]. In the literature, the frost hardiness increases when the water content in one-year-old branches or parts from other organs/tissues (including bark, roots, and buds) of walnut trees decreases [20]. As one study described, tree age influenced bark moisture content; the average moisture content in old trees was lower than that in younger trees [21]. The economic value of rubber trees is predominantly obtained from their bark. Bark is also subject to latex bleeding triggered by chilling stress. Therefore, by establishing the correlation between the chilling resistance of rubber trees and the bark-bleeding characteristics caused by chilling stress, considering the physiological indicators, cell wall chemical components, fiber morphologies, and tensile properties of rubber tree bark, we can gain a deeper comprehension of the mechanism that contribute to *Hevea brasiliensis* chilling resistance and bark bleeding under chilling stress. Understanding this relationship will help inform recommendations for preventing bark bleeding and cold damage in rubber trees and provide a scientific basis for bark-bleeding mechanisms in rubber trees under chilling stress.

## 2. Results

### 2.1. Differences in Physiological Parameters, Chemical Components, Fiber Morphologies, and Tensile Properties in Bark of Hevea brasiliensis at Different Ages

#### 2.1.1. Differences in Chlorophyll Content in Bark of *Hevea brasiliensis* at Different Ages

**Figure 1:**
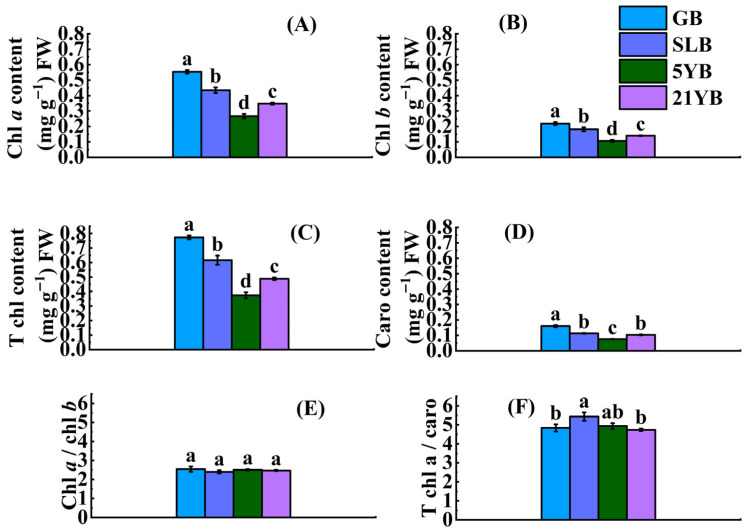
Chlorophyll content in GB, SLB, 5YB, and 21YB. (**A**) Chlorophyll *a* content. (**B**) Chlorophyll *b* content. (**C**) Total chlorophyll content. (**D**) Carotenoid content. (**E**) The ratio of chlorophyll *a*/chlorophyll *b*. (**F**) The ratio of total chlorophyll/carotenoid content. The data represent the average of three replicates. The bars show the standard errors. The different lowercase letters indicate significant differences (Duncan’s test of one-way ANOVA, *p* < 0.05) among the statistical data. FW means the fresh weight.

Tree bark supplies a certain amount of photosynthetic surface, and its photosynthetic pigment content varies at different phases of a tree’s lifecycle. Significant differences (*p* < 0.05) were observed in the contents of chlorophyll a (chl *a*), chlorophyll *b* (chl *b*), and total chlorophyll (T chl) among green bark (GB), semi-lignification bark (SLB), and 2-year-old twig bark from 5-year-old rubber trees (5YB) and from 21-year-old rubber trees (21YB). The results of ANOVA showed that the chlorophyll content in GB was significantly higher (*p* < 0.05) than that in the others (Figure 1A–D). Compared to the GB samples, the SLB samples exhibited a 21.59% reduction in chl *a* content, while the 5YB and 21YB samples showed decreases of 51.78% and 37.17%, respectively (Table S1). In GB, the chl *b* content was 0.22 mg g^−1^, and this value was significantly higher (*p* < 0.05) than that in SLB, 5YB, and 21YB (Table S1). The T chl content in bark at different ages varied from 0.37 to 0.77 mg g^−1^ (Table S1). The caro content in SLB was similar to that in 21YB, so there was no significant difference between them. Aside from the discrepancy in caro content between SLB and 21YB, the differences in caro content among all other pairs were statistically significant (*p* < 0.05). When comparing the chlorophyll content, only minor variability was observed in the chl *a*/chl *b* ratio. The ratios of chl *a*/chl *b* in the samples oscillated from 2.40 to 2.54 (Table S1). Compared with the GB, the differences in chl *a*/chl *b* ratios among SLB, 5YB, and 21YB were not significant (Figure 1E). The T chl/caro ratios in GB, SLB, 5YB, and 21YB were approximately twice as high as the corresponding chl *a*/chl *b* ratios within these samples, and the values ranged from 4.73 to 5.44 (Table S1). Meanwhile, the T chl/caro ratio in SLB exhibited the greatest disparity compared to that in GB, 5YB, and 21YB. With the exception of the T chl/caro ratio in 5YB, the ratio in SLB was significantly higher (*p* < 0.05) than that in GB and 21YB (Figure 1F). Then, the comprehensive evaluation values of photosynthetic pigment content (chl *a*, chl *b*, Tchl, and caro) in the samples were ranked, based on their numerical values in Table S2. The comprehensive values in GB, SLB, 5YB, and 21YB were 1, 0.58, 0, and 0.30, respectively. This elucidated that the photosynthetic activity in the four types of bark increased in the order GB > SLB > 21YB > 5YB.

#### 2.1.2. Differences in RWC in Bark from *Hevea brasiliensis* at Different Ages

**Figure 2:**
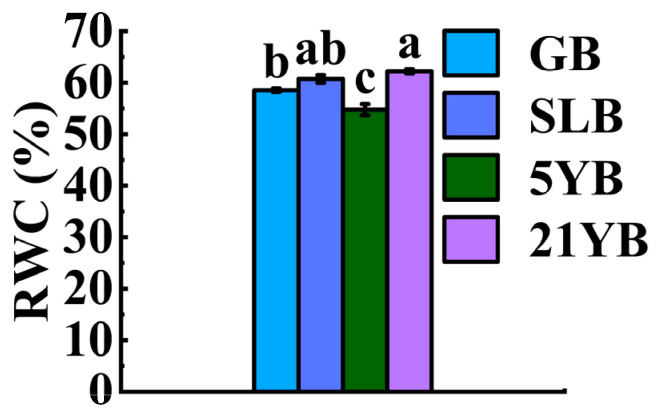
Changes in the RWC in rubber tree bark at different ages. The data represent the average of three replicates. The bars show the standard errors. The different lowercase letters indicate significant differences (Duncan’s test of one-way ANOVA, *p* < 0.05) among the statistical data.

More and more studies have shown that tree age influences bark water content. Meanwhile, the water content in bark varies widely and is affected by many factors. According to Figure 2, the RWC value in 21YB was higher than that in GB, SLB, and 5YB. Except for the value in SLB, it was obviously (*p* < 0.05) higher than all of them (Table S3).

#### 2.1.3. Differences in Chemical Components in Cell Walls of *Hevea brasiliensis* Bark at Different Ages

**Figure 3:**
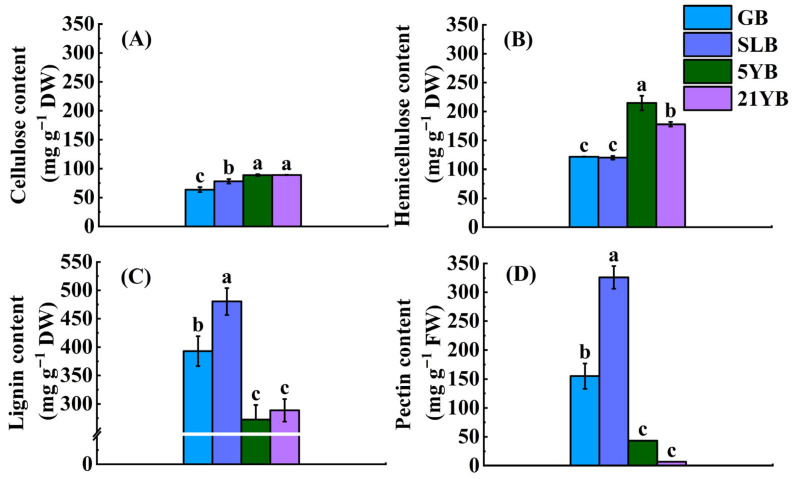
Chemical component content in GB, SLB, 5YB, and 21YB. (**A**) Cellulose content. (**B**) Hemicellulose content. (**C**) Lignin content. (**D**) Pectin content. The data represent the average of three replicates. The bars show the standard errors. The different lowercase letters indicate significant differences (Duncan’s test of one-way ANOVA, *p* < 0.05) among the statistical data. FW means fresh weight. DW means dry weight.

In general, tree age also influences the chemical component content in bark. As rubber trees age, the cellulose content in the bark increases accordingly (Figure 3A). 21YB had the highest cellulose content, which was higher than that in GB, SLB, and 5YB by 39.69%, 13.70%, and 0.33%, respectively (Table S4). With the exception of the minimal variance in cellulose content between 5YB and 21YB, the discrepancies in cellulose content among other pairs were statistically significant (*p* < 0.05) (Figure 3A). With the increase in the tree age, the hemicellulose content in the bark showed a fluctuating trend of first decreasing, then increasing, and finally decreasing again (Figure 3B). The hemicellulose content in 5YB was visibly higher (*p* < 0.05) than that in other individuals. As the data denoted, the hemicellulose content in GB was approximately equal to the value in SLB, so no difference between the values in GB and SLB was found. In the case of the lignin content, its value in SLB was 1.22 times that in GB, 1.76 times that in 5YB, and 1.66 times that in 21YB (Table S4). No significant difference between the lignin content in 5YB and 21YB was observed (Figure 3C). As Table S3 indicates, the pectin content in SLB was significantly higher than that in GB, 5YB, and 21YB. Specifically, it was 1.10-fold, 6.52-fold, and 46.25-fold greater (Table S4), respectively. With the exception of the difference in pectin content in 5YB and 21YB, the differences in pectin content between other pairs reached significant (*p* < 0.05) levels (Figure 3D).

#### 2.1.4. Differences in Fiber Dimensions in Cell Walls of *Hevea brasiliensis* Bark at Different Ages

**Figure 4:**
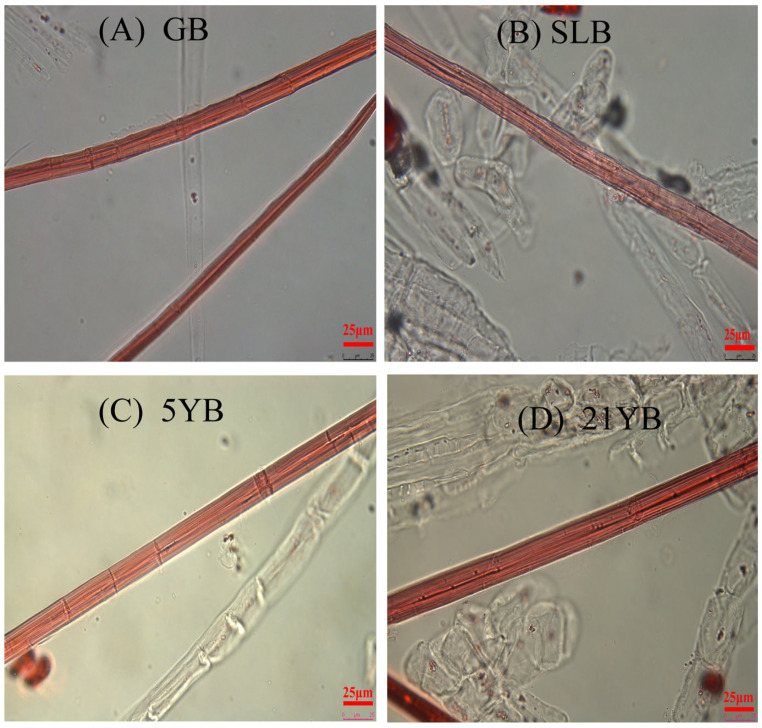
Fiber morphologies in bark of seedling stems and twig segments. (**A**) Fiber morphology in GB. (**B**) Fiber morphology in SLB. (**C**) Fiber morphology in 5YB. (**D**) Fiber morphology in 21YB.

Bark age is also vital when considering its inherent growth, because it is known that age-related increases in bark are accompanied by structural changes. As Table 1 and Figure 4 depict, FL differed visibly, and it increased gradually with age. The average FL ranged from 2722.37 μm to 4014.67 μm; 21YB had the highest mean FL, and GB had the lowest mean FL. The mean FW in GB, SLB, 5YB, and 21YB was 19.91 μm, 18.87 μm, 22.18 μm, and 20.80 μm, respectively. Consequently, 5YB had the highest FW, which was obviously higher (*p* < 0.05) than that of other tree kinds of bark. As the statistical data denoted, the DWT showed an analogous variation trend with the FW, and the value in 5YB was the highest among them. On the whole, the LD decreased slightly with age, and the value varied from 1.13 μm to 1.32 μm. There were no significant differences among them. For the FL/FW ratio, 21YB showed the highest value, and it was significantly higher (*p* < 0.05) than the others. Meanwhile, the FL/FW ratio in 5YB was also obviously higher (*p* < 0.05) than that in GB. From Table 1, the mean DWT/LD ratio in GB, SLB, 5YB, and 21YB varied from 16.54 μm to 19.11 μm. The value in 5YB was the biggest, and markedly bigger (*p* < 0.05) than that in GB and SLB. But the difference in the DWT/LD ratio between 5YB and 21YB failed to reach significance. In terms of the LD/FW ratio, SLB had the highest value, and 5YB had the lowest value. The LD/FW ratio in SLB was statistically higher (*p* < 0.05) than that in 5YB and 21YB.

#### 2.1.5. Differences in Tensile Properties in *Hevea brasiliensis* Bark at Different Ages

The results of the tensile testing are exhibited in Table 2. According to the testing, 17 GB specimens, 22 SLB specimens, 15 5YB specimens, and 11 21YB specimens were accomplished in the tensile assignments. Bark thickness (BT) values ranged from 0.6271 mm to 1.0993 mm among bark from rubber trees at different ages. As shown in Table 2, the BT values in 5YB and 21YB were both significantly larger (*p* < 0.05) than those in GB and SLB, but no significant difference in the values between 5YB and 21YB could be seen. Among the four kinds of bark, 5YB had the highest mean ML, and SLB had the lowest. The maximum load (ML) value in 5YB was 1.23 times that in GB, 1.56 times that in SLB, and 1.03 times that in 21YB, respectively. However, the difference in ML value between 5YB and 21YB was not obvious (*p* < 0.05). The tensile strength (TS) value in GB was 9.6647 MPa on average, which was the highest among the four kinds of bark and obviously higher (*p* < 0.05) than the others. For the elongation at break point (EBP), the mean value varied from 0.28% to 0.34%; there were no significant differences among them. In terms of Young’s modulus (YM), the average values in GB, SLB, 5YB, and 21YB were 3074.6760 MPa, 2677.2310 MPa, 2283.2720 MPa, and 2716.7490 MPa, respectively. The statistical results were comparable to those of the EBP.

**Figure 5:**
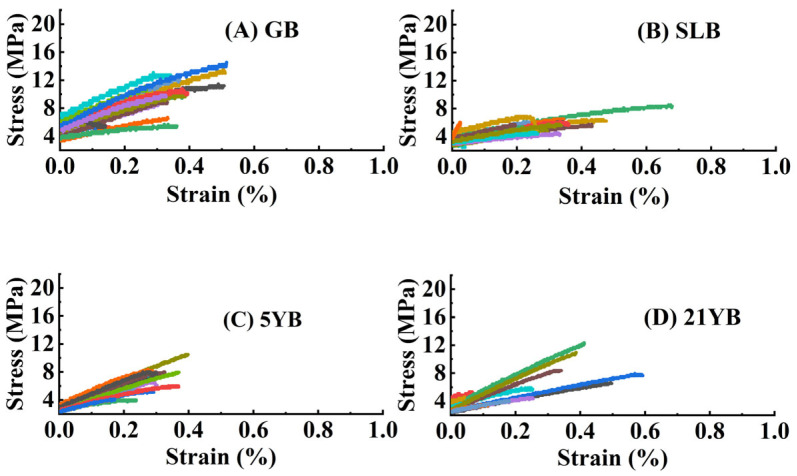
Stress–strain curves for bark in GB, SLB, 5YB, and 21YB. Different colors represent different stress–strain curves of different successful testing specimens, respectively. (**A**) Stress–strain curves in GB. (**B**) Stress–strain curves in SLB. (**C**) Stress–strain curves in 5YB. (**D**) Stress–strain curves in 21YB.

The tensile mechanical behavior in the bark of the rubber trees at different ages is presented in Figure 5. From Figure 5, the results of tensile deformation in different specimens could be observed. The curves in Figure 5 illustrate that the tensile deformation behavior of bark was similar to that of general fibers to a certain degree. The stress–strain behavior was elastic, followed by yielding, linear strain-hardening, and, at last, an ultimate fracture at the breaking point.

### 2.2. Changes in Relative Electrical Conductivity (REC) Under Low-Temperature Stresses and Semi-Lethal Temperature (LT_50_) Estimation

**Figure 6:**
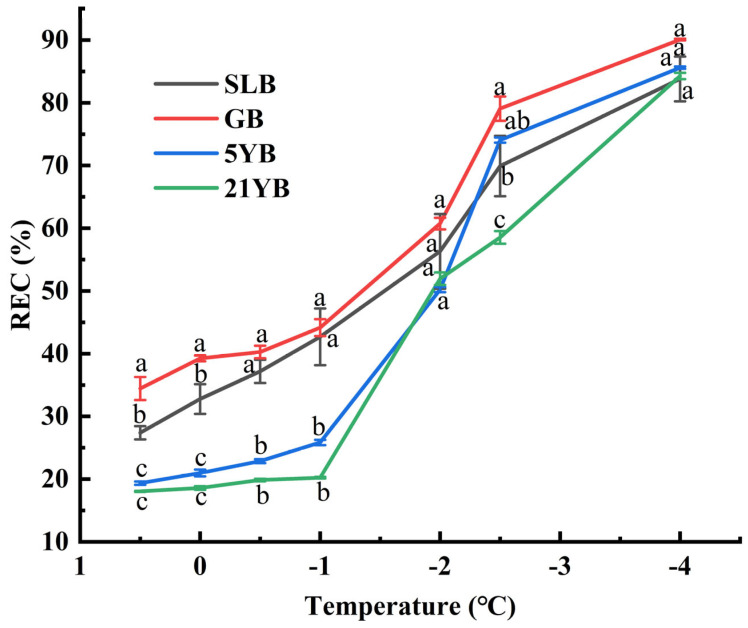
REC values in bark from rubber tree seedlings and twig segments at different ages under different low temperatures. The data represent the average of three replicates. The bars show the standard errors. The different lowercase letters at each chilling-stress temperature indicate significant differences (Duncan’s test of one-way ANOVA, *p* < 0.05) among the statistical data.

As shown in Figure 6, during exposure to low-temperature stress, the REC values in bark at different ages in rubber trees increased gradually. This increase was in line with decreasing temperatures, suggesting a negative correlation between the REC and the temperature. As the temperature decreased, the damage to cell membrane permeability became greater, leading to an increase in electrolyte leakage. However, the amplitudes of REC in different low-temperature changing phases were different. As temperatures decreased from 0.5 °C to −1 °C, the REC values progressively increased, and the REC values in GB, SLB, 5YB, and 21YB increased by 28.17%, 55.85%, 33.54%, and 12.10%, respectively (Table S5). Furthermore, at −1 °C, 5YB and 21YB displayed evidently lower REC values (*p* < 0.05) compared to GB and SLB, indicating that the two maintained better integrity of their cell membranes under this low-temperature condition. The REC values showed the fastest increase during the chilling-stress process from −1 °C to −2.5 °C; the REC values increased by 79.11%, 63.81%, 186.47%, and 189.30%, respectively (Table S5). At −2.5 °C, 21YB exhibited a significantly lower (*p* < 0.05) REC value compared to others, suggesting its stronger physiological adaptations to chilling stress. From −2.5 °C to −4 °C, the REC values slowly changed, indicating a dramatic increase in cell membrane permeability and severe damage to the integrity of the cell membrane. The REC value reached a maximum, but there were no significant differences among them. Overall, the chilling-stress temperature and the REC value followed an asymmetric sigmoid curve.

Logistic regression analysis of the REC data determined the LT_50_ for bark in rubber trees, quantifying their chilling resistance levels (Table 3). According to Table 3, the fitting degree (R^2^) of the LT_50_ equation of bark in rubber tree seedlings and twig segments at different ages was above 0.9341, and it reached extremely significant levels (*p* < 0.01). The LT_50_ values ranged from −2.0387 °C in 21YB to −0.8695 °C in GB, elucidating substantial variability in chilling resistance among bark in rubber tree seedlings and twig segments at different ages. LT_50_ is negatively correlated with plant chilling resistance, and this indicates that the lower the LT_50_, the stronger the cold resistance in plants. The chilling-resistance order of bark in rubber tree seedlings and twig segments at different ages was as follows: 21YB > 5YB > SLB > GB.

### 2.3. Stem Age-Mediation of Seedling Responses to Chilling Stress

#### 2.3.1. Changes in Chlorophyll Content in Stem Bark of *Hevea brasiliensis* Seedlings at Different Stem Ages Under Chilling Stress

**Figure 7:**
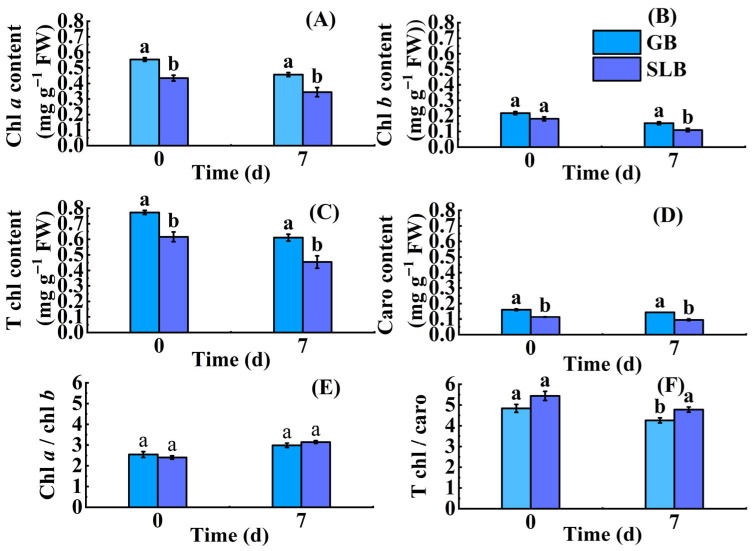
Changes in chlorophyll content in GB and SLB in rubber tree seedlings under chilling stress. (**A**) Chlorophyll *a* content. (**B**) Chlorophyll *b* content. (**C**) Total chlorophyll content. (**D**) Carotenoid content. (**E**) The ratio of chlorophyll *a*/chlorophyll *b*. (**F**) The ratio of total chlorophyll/carotenoid content. The data represent the average of three replicates. The bars show the standard errors. The different lowercase letters indicate significant differences (Duncan’s test of one-way ANOVA, *p* < 0.05) among the statistical data. FW means fresh weight.

After 7 days of low-temperature stress, the contents of chlorophyll *a* (chl *a*), chlorophyll *b* (chl *b*), total chlorophyll (T chl), and carotenoid (caro) in GB and SLB were all lower than those before the stress (Figure 7A–D). However, the reductions in chl *a* content, chl *b* content, T chl content, and caro content in GB were 17.49%, 29.77%, 20.96%, and 10.49%, respectively. These decreased values were all less than those in SLB, which were 20.72%, 39.63%, 26.28%, and 16.34% (Table S6). As shown in the data, the chl *b* content was most affected by low-temperature stress, while the caro content was the least. The chlorophyll content in GB was higher than that in SLB both before and after low-temperature stress. There were significant differences in chlorophyll (*p* < 0.05) content between GB and SLB, except for chl *b* content before the low-temperature stress. Following a 7-day period of chilling stress, there was a slight increase in the chl *a*/chl *b* ratios in GB and SLB, with enhancements of 17.40% and 30.97% (Table S6), respectively, relative to their pre-stress levels (Figure 7E). The ratio of chl *a* /chl *b* in GB was higher than that in SLB before chilling stress. This was opposite to that after chilling stress (Figure 7E). However, no significant differences (*p* < 0.05) were observed between chl *a*/chl *b* ratios in GB and that in SLB before and after chilling stress (Figure 7E). In contrast to the ratio of chl *a*/chl *b*, the ratio of T chl/caro in GB and SLB diminished slightly after the 7-day stress (Figure 7F). They declined 11.96% and 12.05% (Table S6), respectively, compared to their pre-stress levels. Although the ratio of T chl/caro in GB was higher than that in SLB before and after chilling stress, the difference between them was statistically significant (*p* < 0.05) only after the stress.

#### 2.3.2. Changes in RWC in Stem Bark of *Hevea brasiliensis* Seedlings at Different Stem Ages Under Chilling Stress

**Figure 8:**
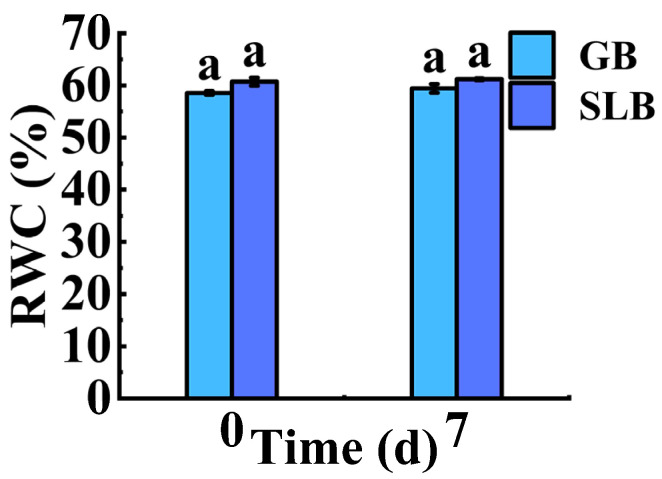
Changes in the RWC in GB and SLB in rubber tree seedlings under chilling stress. The data represent the average of three replicates. The bars show the standard errors. The different lowercase letters indicate significant differences (Duncan’s test of one-way ANOVA, *p* < 0.05) among the statistical data.

There is a causal relationship between bark water content and the chilling tolerance of trees. After the 7-day chilling stress, the RWC values in GB and SLB increased by 0.79% and 1.54% of their initial values (Table S7), respectively. These changes were small and not pronounced (Figure 8). After the chilling stress process, the difference in the RWC values between SLB and GB decreased from 3.74% to 2.97% (Table S7). Accordingly, the discrepancy was still non-significant after chilling stress (Figure 8).

#### 2.3.3. Changes in Chemical Components in Stem Bark of *Hevea brasiliensis* Seedlings at Different Stem Ages Under Chilling Stress

**Figure 9:**
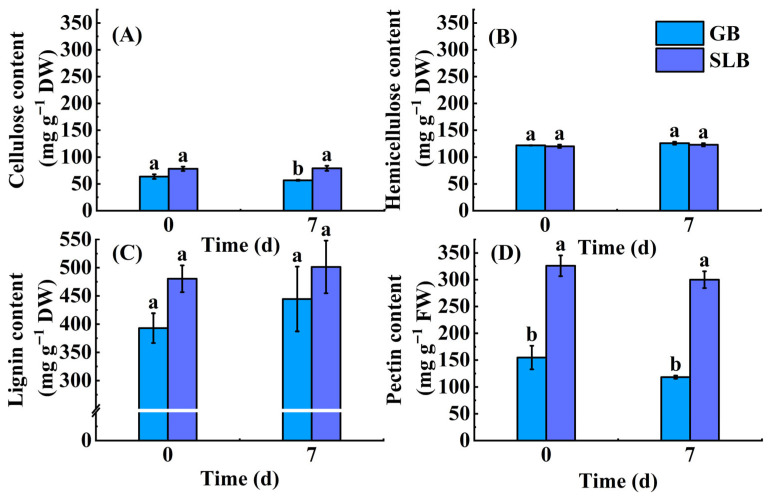
Changes in the chemical component content of the cell walls in GB and SLB in rubber tree seedlings under chilling stress. (**A**) Cellulose content. (**B**) Hemicellulose content. (**C**) Lignin content. (**D**) Pectin content. The data represent the average of three replicates. The bars show the standard errors. The different lowercase letters indicate significant differences (Duncan’s test of one-way ANOVA, *p* < 0.05) among the statistical data. FW means fresh weight. DW means dry weight.

Changes in the chemical components in the bark of seedlings during chilling stress were depicted in Figure 9A–D. As can be observed in Figure 9A, the cellulose content in GB decreased by 10.82%, and in SLB increased by 1.15% (Table S8) after the end of the chilling stress. Although the cellulose content in SLB was always higher than that in GB in the non-chilling phase and the termination of chilling stress, a significant difference (*p* < 0.05) was only found between them after chilling stress. As Figure 9B demonstrates, the hemicellulose content in GB and SLB slightly increased as a result of chilling stress. During chilling stress, the hemicellulose content increased by 3.37% and 2.35% (Table S8) above non-stress values in GB and SLB, respectively. GB had small higher hemicellulose content than SLB before and after the stress, whereas the values did not differ between them. Upon analyzing the samples from GB and SLB submitted to chilling stress, an increase in lignin content of 13.13% and 4.38% (Table S8) was found (Figure 9C). In comparison to GB, SLB demonstrated an increase in lignin content by 22.26% before the stress and 12.81% after the stress (Table S8), but there were no significant differences between them consistently. Contrary to the lignin content, at the end of the chilling-stress period, the pectin content was decreased by 7.93% in SLB and 23.61% in GB, respectively (Table S8). Under chilling stress conditions, SLB showed a 1.54-fold (Table S8) increase in pectin content compared to that in GB, and there was a significant discrepancy (*p* < 0.05) between them (Figure 9D). This was also the case before the stress was applied, with a 1.10-fold increase in pectin content (Table S8).

#### 2.3.4. Changes in Fiber Dimensions in Stem Bark of *Hevea brasiliensis* Seedlings at Different Stem Ages Under Chilling Stress

**Figure 10:**
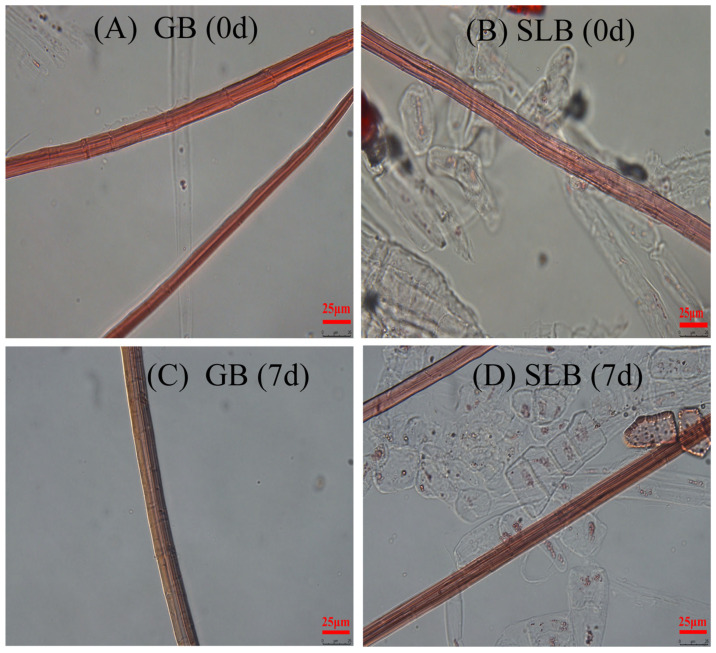
Fiber morphologies in bark from seedling stems under chilling stress. (**A**) The fiber morphology in GB before chilling stress. (**B**) The fiber morphology in SLB before chilling stress. (**C**) The fiber morphology in GB after the 7-day chilling stress. (**D**) The fiber morphology in SLB after the 7-day chilling stress.

Fiber length (FL), fiber width (FW), double-wall thickness (DWL), lumen diameter (LD), fiber length/fiber width (FL/FW), double-wall thickness/lumen diameter (DWT/LD) and lumen diameter/fiber width (LD/FW) in stem bark in rubber tree seedlings are listed in Table 4. Meanwhile, fiber morphologies are exhibited in Figure 10. As shown in Table 4, the FL exhibited a significant difference (*p* < 0.05) in response to the chilling-stress treatment. Increases in FL were observed at 15.11% and 12.73% for GB and SLB, respectively, compared to their pre-stress levels. After chilling stress, the FW values in GB and SLB were smaller compared with those before the stress, while there was a distinct difference (*p* < 0.05) only in SLB before and after the stress. The chilling treatment had a significant influence on the DWT in GB and SLB. Accordingly, the value of the DWT in GB and SLB decreased by 8.20% and 6.99%, respectively. Variations in the fiber LD in GB and SLB exhibited contrary trends under chilling stress. This value in GB increased markedly (*p* < 0.05) from non-stress conditions to chilling-stress conditions, but that in SLB decreased slightly. The FL/FW ratios in GB and SLB increased obviously (*p* < 0.05) with chilling treatment, with enhancements of 19.76% and 19.86%, respectively. Contrary to the FL/FW ratios, the fiber DWT/LD ratios in GB and SLB decreased distinctly (*p* < 0.05) under chilling treatment. As shown in Table 4, the LD/FW ratios in GB and SLB were higher than those before the stress. However, no significant difference in the LD/FW ratio in SLB was found before and after the stress.

#### 2.3.5. Changes in Tensile Properties in Stem Bark of *Hevea brasiliensis* Seedlings at Different Stem Ages Under Chilling Stress

Overall, 17 GB specimens and 22 SLB specimens were successfully tested and analyzed before chilling stress. After the stress, the number of successful testing specimens in GB and SLB was 14 and 15, respectively. The bark thickness (BT) in GB and SLB decreased during the 7-day chilling stress. The BT in SLB after chilling stress was significantly less (*p* < 0.05) than that before the stress, whereas the BT in GB was only slightly lowered. The results in Table 5 indicate that the maximum load (ML) in GB and SLB slightly increased with chilling stress. However, the ML values in GB were pronounced higher (*p* < 0.05) than those in SLB before and after the stress. Regardless of chilling stress, the tensile strength (TS) in GB was always significantly higher (*p* < 0.05) compared with that in SLB. Compared to SLB, the TS in GB was obviously (*p* < 0.05) enhanced with chilling stress, though the enhancements of the TS value in GB and SLB were 21.83% and 28.40%, respectively, after the stress. The mean elongation at break point (EBP) in GB under chilling stress was greatly higher (*p* < 0.05) compared with that in SLB. As Table 5 illustrates, the EBP values in GB were 1.20 times and 2.08 times that in SLB before and after the stress, respectively. Furthermore, the EBP value in GB under stress was significantly greater (*p* < 0.05) than that in itself before the stress. The mean value for Young’s modulus (YM) in GB was the highest with chilling stress, but there were no visible differences in YM values between GB and SLB.

**Figure 11:**
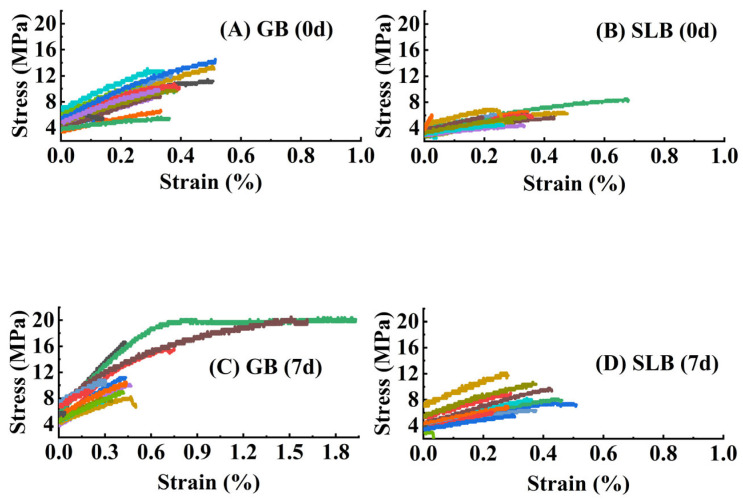
Stress–strain curves for bark in GB and SLB under chilling stress. The different colors represent the different stress–strain curves of the different successful testing specimens, respectively. (**A**) The stress–strain curves in GB before chilling stress. (**B**) The stress–strain curves in SLB before chilling stress. (**C**) The stress–strain curves in GB after the 7-day chilling stress. (**D**) The stress–strain curves in SLB after the 7-day chilling stress.

The stress–strain curves obtained from the stem bark in rubber tree seedlings are exhibited in Figure 11. The overall tensile process for bark specimens could be divided into two phases. In the initial phase, tensile deformation experienced a significant increase in response to tensile force. Subsequently, the increasing trend decelerated until tensile failure occurred.

### 2.4. Results of Bark Bleeding

**Figure 12:**
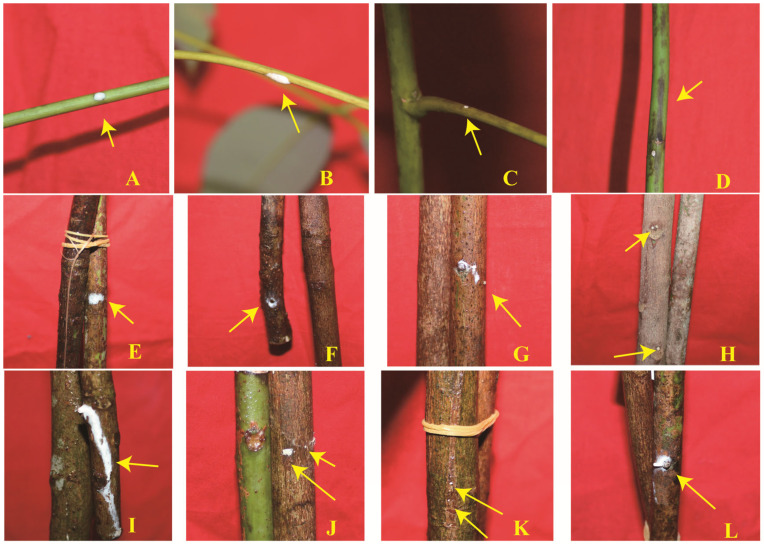
Visual bark bleeding in bark in rubber tree seedlings and twig segments. (**A–D**) 1-year-old seedlings. (**E**–**H**) 2-year-old twig segments in 5-year-old rubber trees. (**I**–**L**) 2-year-old twig segments in 21-year-old rubber trees.

The symptom of bark bleeding emerged in rubber tree seedlings after 2 days of stress, and the symptom emerged in twig segments after 1 day of stress. Symptomatic seedlings exuded latex on their petioles (Figure 12A–C), which were accompanied by the emergence of dark spots on the stems (Figure 12D). However, twigs exuded latex on bundle scars (Figure 12F–I,L) and lenticels (Figure 12E,J,K). The visual records and statistical data are illustrated in Table 6. As Table 6 illustrates, the performance of bark bleeding among seedlings and twig segment bark in rubber trees at different ages was distinct. The syndrome of bark bleeding in SLB in seedlings was not observed. Meanwhile, the number of bark-bleeding positions (NBPs) and the area of bark bleeding (AB) in 5YB were both the highest. Moreover, the bark-bleeding symptom appeared in all twig segments in 5YB and 21YB.

### 2.5. A Comprehensive Analysis of Chilling Resistance and Bark-Bleeding Characteristics in Bark at Different Ages in Rubber Trees

**Figure 13:**
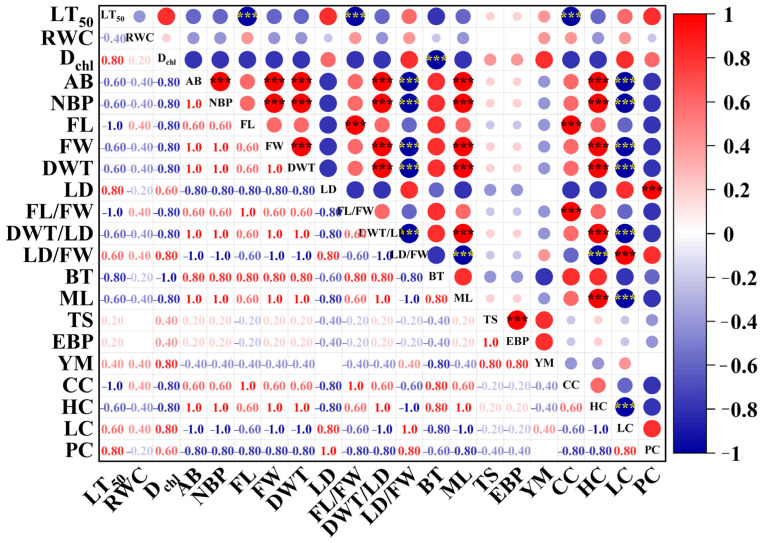
Correlation analysis between the LT_50_, bark bleeding in seedlings and twig segments exposed to chilling stress, RWC, D_chl_, cell-wall chemical components, fiber characteristics, and bark tensile properties of bark at different ages in rubber trees. LT_50_, the semi-lethal temperature. RWC, the relative water content. D_chl_, the comprehensive evaluation value of pigment indicators. AB, the area of bark bleeding. NBPs, the number of bark-bleeding positions. FL, the fiber length. FW, the fiber width. DWT, the fiber double-wall thickness. LD, fiber lumen diameter. FL/FW, the ratio of fiber length to fiber width. DWT/LD, the ratio of fiber double-wall thickness to fiber lumen diameter. LD/FW, the ratio of fiber lumen diameter to fiber width. BT, bark thickness. ML, the maximum load. TS, tensile strength. EPB, the elongation at the break point. YM, Young’s modulus. CC, the cellulose content. HC, the hemicellulose content. LC, the lignin content. PC, the pectin content. The larger the circle and the darker the color, the larger the correlation coefficient. Negative correlations denoted in blue and positive correlations denoted in red. *** *p* < 0.001.

The LT_50_ value accurately interpreted the chilling resistance of bark at different ages in rubber trees, so it can be used as the chilling-resistance trait of bark. Then, the LT_50_ was integrated with the bark-bleeding statistical data, RWC values, bark cell-wall chemical component contents, fiber dimensions, and bark mechanical property parameters to create a Spearman correlation matrix (Figure 13). It was a convenient way to ascertain the relationships among these variables. From the results, LT_50_ was highly negatively (*p* < 0.001) correlated with the fiber length (FL), the ratio of fiber length to fiber width (FL/FW), and cellulose content (CC). The comprehensive evaluation value of pigment indicators (Dchl) was markedly negatively related to the bark thickness (BT). The area of bark bleeding (AB) was significantly positively correlated (*p* < 0.001) with the number of bark-bleeding positions (NBPs), fiber width (FW), double-wall thickness (DWT), ratio of double-wall thickness to lumen diameter (DWT/LD), maximum load (ML), and hemicellulose content (HC). Furthermore, the area of bark (AB) was significantly negatively related (*p* < 0.001) to the ratio of the fiber lumen diameter to the fiber width (LD/FW) and the lignin content (LC). Also, the number of bark-bleeding positions (NBPs) recorded an extremely positive correlation (*p* < 0.001) with the fiber width (FW), double-wall thickness (DWT), ratio of double-wall thickness to the lumen diameter (DWT/LD), maximum load (ML), and hemicellulose content (HC). On the contrary, the number of bark-bleeding positions (NBPs) displayed a strong negative correlation (*p* < 0.001) with the ratio of the fiber lumen diameter to the fiber width (LD/FW) and lignin content (LC). The obviously positive (*p* < 0.001) relationship between the lumen diameter (LD) and the pectin content (PC) also could be found.

## 3. Discussion

The total chlorophyll content (Chl*a*+*b*) in 1-year-old *Ginkga biloba* branches was lower than that in the current year stems [22]. With the 1- and 3-year-old lilal stems aging, the chl *a* and chl *b* content in bark was reduced, but the ratio of chl *a* to chl *b* decreased only slightly [23]. Similar results were obtained in this study, but the photosynthetic pigment content in 5YB was significantly lower (*p* < 0.05) than that in the others, and the ratio chl *a* to chl *b* was slightly higher than that in SLB and 21YB. This might be due to the more vigorous growth of the 5-year-old rubber trees, resulting in a higher canopy density in the 5-year-old rubber tree plot and the shading of twigs. This result confirmed the rule that shade-adapted cells and tissue had a lower chl *a* to chl *b* ratio than high-light-adapted cells and tissue [17]. In previous research, the ratio of T chl to caro decreased significantly with the age of the lilal stems [23]. In our results, the Tchl/caro ratio in SLB was the highest, and the chl *a*/chl *b* ratio was the lowest compared to the others. Based on this result, we assumed that SLB met the general criteria for shade tolerance [24]. Tran Trang T et al. (2018) also found *Fraxinus latifolia* seedlings exhibited shade tolerance characteristics due to a decrease in the chl *a*/chl *b* ratio and an increase in the Tchl/caro ratio in the stem bark in *Fraxinus latifolia* seedlings [25]. Pfanz et al. (2002) documented that thicker bark may offer enhanced protection, yet it simultaneously restricts light penetration and decreases the photosynthetic activity [26]. This fact, along with our results observed in our correlation analysis, shows that D_chl_ exhibited an extremely negative (*p* < 0.001) relationship with bark thickness.

Previous studies have demonstrated that tree age influences bark water content. The mean water content in spruce bark was 10.4−13.2% lower in older trees (70 years) compared to younger trees (35 and 55 years) [21]. These findings are not entirely consistent with our study. This is not surprising. As reported in previous studies, the water content in bark is generally able to change and is affected by various factors, such as the tree species, season, and debarking method [27]. We propose that the rubber tree is species-specific, as previous research reported that thick-barked species have copious latex yield, probably because they possess numerous laticifers in their inner bark [28]. Meanwhile, according to rubber tree cultivation biology, 21-year-old rubber trees are in their peak growth and yielding period [1], so 21YB exhibited the highest RWC value in this study.

In our results, changes in chemical component content in the bark of seedlings and twig segments at different ages varied distinctly. As reported in previous studies, the chemical composition of bark varies due to its heterogeneous structure, which is influenced by various factors, including growing conditions, age, region, and the method of sampling [29]. Similarly, Özgenç (2017) also found that the contents in the main cell wall components of the tree barks exhibited variability based on species, growth conditions, regions, and age [30]. To date, there is relatively limited literature on the changing morphological characteristics of bark fibers considering age, and these studies mainly focus on the variation trends in bark fiber morphology from the base to the top of the tree stem [31,32]. Cabalová et al. (2021) found that the average bark fiber length and width increased from the bottom to the top part of the Norway spruce trunk [31]. This agrees with Kathirselvam et al. (2019) observations for bark fibers in the *Thepesia populnea* trunk [32]. Distinct from previous work, bark fiber morphologies in seedling stems and twig segments at different ages demonstrated their specific characteristics. We assumed the fiber structure and composition differ according to the species, and these inherent differences would influence their mechanical properties. This speculation was similar to the results in the literature [33]. Previous research has documented that the mechanical characteristics of plant tissues are predominantly influenced by the spatial organization of fibers within the cell walls [34]. It has been reported that the thickness of tree bark increases proportionally with the age of the tree [35]. Our findings were basically consistent with this finding. This trend could highlight bark thickness as an independent functional trait in plants [36]. Currently, only a few studies concern the variation in bark tensile properties with age. In our study, GB showed the highest TS, EBP, and YM values, perhaps because the fibers in GB (with larger LD values and thinner DWT values) had better interweaving properties with adjoined fibers. These findings are similar to those from Bold et al. (2020), who reported that the process of combining single fibers into fiber pairs does not detrimentally affect their extensibility (as evidenced by no significant difference in maximum strain) but it does notably enhance other mechanical properties, such as the tensile strength, Young’s modulus, and energy dissipated [37].

The previous literature has suggested that trade-offs exist between chilling resistance and the ability to accumulate biomass, which might devote more to chilling resistance and less to developmental reproduction as they age [6]. For this reason, the chilling-resistance order of bark at different ages in the rubber tree follows the result in this study. This is also supported by Mcnamara et al. (2000), who found that wild-growing seedlings of several woody perennials are typically not as chilling resistant as physiologically mature individuals [38]. Previous research has documented that the chloroplast is sensitive to low temperatures because low temperatures depress the metabolism of plants, affecting the biosynthesis of photosynthetic pigments. The decrease in photosynthetic pigments under chilling stress is attributed to stress-induced damage in pigment degradation [39]. In line with these findings, in the present study, the decrease in photosynthetic pigment content in the stem bark in seedlings can be observed. There is more and more consensus that as a tree ages, the increase in bark thickness enhances insulation, making the vascular cambium less susceptible to extreme cold damage [40]. On the other hand, as Rosell et al. (2013) reported, an increase in bark thickness significantly enhanced the stiffness of stems and facilitated greater water storage [19]. These findings align with the fundamental understanding that SLB had higher BT values than GB, so SLB exhibited better chilling resistance and higher RWC than GB. As previously explained, the water content in bark can change in the air’s relative humidity because of bark hygroscopicity [41]. In this experiment, in order to simulate a chilling damage environment, we set the air relative humidity to 80% in the artificial climatic laboratory. We speculated that the stems of the seedlings were saturated with the air’s humidity after the 7-day chilling stress, thus the RWC in the bark of the seedlings increased after the stress.

Many studies have suggested that chilling stress promotes the accumulation of dicotyledons’ cell wall components, including hemicellulose and pectin, which induce tissue and/or organ tensile strength and rigidity and cell wall thickness, thus protecting their cells from chilling damage [42]. In addition, an increase in the lignin content in shoots of young poplar trees was observed after two weeks of chilling stress [43]. Approximately similar results were obtained in this study. Based on our results, we speculated that the pectin was covalently bound to cellulose, different from that in the aforementioned literature. This speculation was similar to the result of previous research [42]. Contrary to our study, it was reported that low temperatures reduced the fiber length significantly in chilling-tolerant and chilling-sensitive cotton cultivars [44]. Perhaps there are species-specific fiber structure and composition changes and differences among individual seedlings. Meanwhile, these intrinsic discrepancies affect their biomechanical properties. The enhancement of Young’s modulus in carrot taproot tissue during cold acclimation demonstrated that the cold acclimation promoted tissue rigidity in plants [45]. The increase in tensile stiffness in oil-seed rape leaves under cold acclimation was associated with increased CDTA-soluble pectin content in the cell walls [46]. Collectively, these findings underscore that Young’s modulus in plant tissues and organs increased under low temperatures. This supports our results. Unlike occurrence sites in rubber trees caused by pests, diseases [4,47], and other abiotic stresses, this symptom emerged on petioles in seedlings and on bundle scars and lenticels in twig segments, which is similar to that of peach gummosis [48]. Based on our results, the chilling resistance of 5YB and 21YB twig segments was significantly stronger than that of seedlings. On the contrary, the rates of bark-bleeding in biennial twig segments (RBTSs) in 5YB and 21YB were higher than the rate of bark-bleeding in seedlings (RBSs). This was likely due to the increased susceptibility of twig segments with pruning wounds under chilling stress. Interestingly, in our previous experiment, 1-year-old budded stump seedlings emerged with bark bleeding on their tender stems and leaf scars [49]. This divergence likely stems from the variations in seedling varieties and types of budding.

As the previous literature has described, the trade-offs and the coordination among the various functions in bark represent significant yet often neglected factors that influence the ecology and evolutionary development of bark [19]. This agrees with Pásztory et al. (2016), who reported that bark served multiple functions throughout the lifespan of a plant, and its characteristics evolve with age [27]. Based on our statistical data, the significant correlations between bark function–trait parameters appear to be more suitable for supporting the literature. These results can better help us comprehend the chilling resistance of rubber trees and the mechanisms of bark bleeding caused by chilling stress. However, it is crucial to recognize that this relationship is not consistently straightforward. Thus, further studies are needed to reveal the changes in these function–trait parameters under chilling stress and the occurrence of bark bleeding from a microscopic perspective. Meanwhile, further research should investigate the synergistic mechanisms underlying these functions in bark.

## 4. Materials and Methods

### 4.1. Experimental Materials

**Figure 14:**
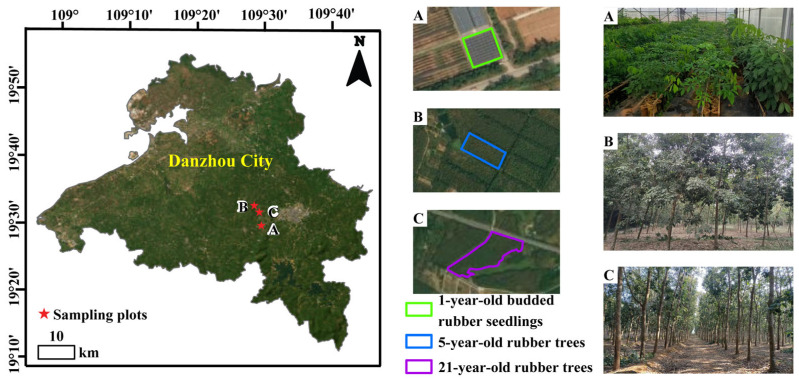
General location of the study areas. Left, the location of the study area and the three sampling plots of the rubber trees within the High-Quality Seedling Propagation Nursery of Rubber Research Institute and Experimental Farm of the Chinese Academy of Tropical Agricultural Sciences, Danzhou, Hainan Island, China. Middle, the remote sensing images, where the different colorful figures exhibit the edges of the different sampling plots. Right, the photos show the sampling plot’s detailed locations. (**A**) 1-year-old budded rubber seedlings. (**B**) 5-year-old rubber trees. (**C**) 21-year-old rubber trees.

As shown in Figure 14, 1-year-old budded rubber seedlings with three whorls of leaves were grown in the High-Quality Seedling Propagation Nursery of Rubber Research Institute, Chinese Academy of Tropical Agricultural Sciences (109°29′22″ E, 19°29′36″ N, Figure 14A). Five-year-old (109°28′20′′ E, 19°28′20′′ N, Figure 14B) and twenty-one-year-old rubber trees (109°28′20″ E, 19°32′33″ N, Figure 14C), located in the Experimental Farm of the Chinese Academy of Tropical Agricultural Sciences, were used to provide two-year-old twigs. They are the universally adapted *Hevea* cultivar (Reyan 7-33-97) in China. Two-year-old twigs (30 cm length) from five-year-old and twenty-one-year-old rubber trees were randomly collected midmorning in January from forty-two healthy trees of each age, respectively. A total of 84 twig segments from each age of the trees were cut and the leaves were removed, then placed in buckets with tap water for transport to the laboratory.

### 4.2. Chilling-Stress Treatment and Experiment Design

We conducted two chilling-stress experiments in biochemical incubators at the artificial climatic laboratory. Seedlings (3 seedlings) in each testing temperature environment and twig segments (6 twig segments of 3 individuals at each age of trees in each testing temperature environment) were subjected to chilling stress in biochemical incubators at −4 °C, −2.5 °C, −2.0 °C, −1.0 °C, −0.5 °C, 0 °C, and 0.5 °C. The duration of each testing temperature was 20 h. To prevent desiccation, twig segments were set in plastic bags, sprayed with a small quantity of ultrapure water. After the chilling-stress treatment, the samples were removed from the chemical incubators and used for relative electrical conductivity (REC) measurement. Bark tissues were separated from the xylem using a single-sided blade. Twig segments (30 sample twigs of each age of 15 trees) were placed in buckets with a 1/10 Woody Plant Medium (WPM) solution (sucrose- and agar-free). Subsequently, the seedlings and sample twigs immersed in the WPM solution were exposed to the chilling treatment in the artificial climatic laboratory (16 °C/4 °C, day/night) under a photoperiod of 12 h, relative humidity of 80%, and light intensity of 100 μm m^−2^ s^−1^.

Stem bark from the seedlings was harvested from two parts of stems: stems below the first whorl of leaves that were semi-lignified (SLB) and stems above the first whorl of leaves that were unlignified, green, and tender (GB). On the 0 and 7th day of the chilling treatment, the photosynthetic pigment content [chlorophyll *a* (chl *a*), chlorophyll *b* (chl *b*), Total chlorophyll (T chl), and carotenoid (caro)], relative water content (RWC), chemical component content, tensile properties, and fiber morphology characteristics of the stem bark of the seedlings were determined. At ambient temperature, the photosynthetic pigment content (chl *a*, chl *b*, Tchl, and caro), relative water content (RWC), cell-wall chemical component content, tensile properties, and fiber morphological characteristics of the twig bark were ascertained. In addition, seedling and twig segment phenotypes were observed daily during the chilling treatment, and the representations of bark bleeding were recorded from their emergence to the termination of the treatment.

### 4.3. Electrical Conductivity Measurement

After chilling stress in biochemical incubators, seedling stems and 2-year-old twigs were cleaned with tap water and ultrapure water. Soon afterwards, bark tissues of stems from one-year-old seedlings and from 5-year-old and 21-year-old twigs were cut into 5 mm long sections, avoiding leaf scars and buds, and 0.05 g of each sample was placed in a 50 mL centrifuge tube containing 20 mL ultrapure water. Each assay was replicated three times. The tubes were shaken on a shaker at ambient temperature for 10 min. Following vacuum infiltration for 30 min, the tubes were shaken for 10 min again. After that, let the tubes stand for 30 min. The initial electrical conductivity (EC_initial_) of the sample was measured with a conductivity meter (Mettler Toledo, FE38, Zurich, Switzerland). The samples were then boiled in a water bath for 30 min, and after cooling to ambient temperature, the final conductivity (EC_final_) was determined. The relative conductivity was calculated according to the following Formula (1).

REC (%) = (EC_initial_/EC_final_) × 100 (1)

The value of LT_50_ (the semi-lethal temperature) was calculated using the logistic function (2) [50].

y = k/(1 + ae^−bx^) (2)

where y is the REC, x is the treatment temperature, k represents the maximum leakage rate (100%), and a and b are the function parameters.

### 4.4. Photosynthetic Pigment Content Determination

Each bark section sample (0.10 g) was placed in a 15 mL centrifuge tube containing 15 mL photosynthetic extraction solution (ethanol–acetone–ultrapure water = 4.5:4.5:1). The tubes were shaken for 10 min, away from light, and then incubated in a shady place at ambient temperature for 24 h (shaken several times away from light). Afterwards, absorbance readings of the extraction solution under 470 nm, 645 nm, and 663 nm were obtained using a UV/VIS spectrophotometer (PGENERAL T6 Newcentury, Beijing, China). Details can be referred to in the literature [51].

C*_a_* = 12.72 × *A*_663_ − 2.59 × *A*_645_, (3)

C*_b_* = 22.88 × *A*_645_ − 4.67 × *A*_663_, (4)

C_T_ = C*_a_* + C*_b_*, (5)

Ccaro = (1000 × *A*_470_ − 2.05 × C*_a_* − 114.8 × C*_b_*)/245, (6)

Photosynthetic pigment content (mg g^−1^ FW) = (C × V_T_)/(FW × 1000), (7)

where C*_a_* is the chlorophyll *a* concentration (mg L^−1^), C*_b_* is the chlorophyll *b* concentration (mg L^−1^), C_T_ is the total chlorophyll concentration (mg L^−1^), C_caro_ (mg L^−1^) is the carotenoid concentration (mg L^−1^), and C is the photosynthetic pigment concentration (C*_a_*, C*_b_*, C_T_, and C_caro,_ mg L^−1^). *A*_470_, *A*_645_, and *A*_663_ represent the absorbance of the chlorophyll extract solution at 470 nm, 645 nm, and 663 nm, respectively. FW represents the sample fresh weight (g), and V_T_ (mL) represents the total volume of the extract solution.

### 4.5. Relative Water Content Calculation

The fresh weight (FW) was determined using bark sections from seedling stems and 2-year-old twigs with a balance with an accuracy of 1 mg. Then, sections were killed by oven-drying at 105 °C for 30 min and oven-drying at 80 °C for about 8 h until a constant weight. Sections were weighed again to determine dry weight (DW). The relative water content (%, RWC) was calculated as follows:

RWC (%) = [(FW − DW)/FW] × 100(8)

### 4.6. Cell-Wall Chemical Component Content Quantitation

Bark sections were killed by oven-drying at 105 °C for 30 min and oven-drying at 80 °C for about 8 h, until a constant weight. Then, dry bark sections were ground and sieved through a 40-mesh screen (the diameter of the screen hole i 0.425 mm) to obtain the testing samples for cellulose, hemicellulose, and lignin content quantitation. Fresh bark sections were used for pectin content quantification [52].

The cellulose content of the samples was quantitatively determined using a Cellulose (CLL) Content Assay Kit AKSU007C (Beijing Boxbio Science & Technology Co., Ltd., Beijing, China), according to the manufacturer’s instructions, and three experimental repeats were performed. Cellulose subjected to acidic conditions can be hydrolyzed into β-D-glucose. Subsequently, in a strong acid environment, β-D-glucose undergoes dehydration to produce furfurals. Upon further dehydration and condensation with anthrone, a blue–green furfural derivative is formed, which exhibits a characteristic absorption peak at 620 nm. Furthermore, the extinction coefficient undergoes an alteration.

The hemicellulose content of the samples was quantitatively determined using a Themicellulose Content Assay Kit AKSU008C (Beijing Boxbio Science & Technology Co., Ltd., Beijing, China), according to the manufacturer’s instructions, and three experimental repeats were performed. After acid treatment, hemicellulose is converted into reducing sugars, which then react with 3,5-dinitrosalicylic acid to form a brownish–red amino compound. This product exhibits a characteristic absorption peak at 540 nm, allowing the hemicellulose content to be quantitatively determined from the change in absorbance.

The lignin content of the samples was quantitatively determined using a Lignin Content Assay Kit AKSU010U (Beijing Boxbio Science & Technology Co., Ltd., Beijing, China), according to the manufacturer’s instructions, and three experimental repeats were performed. Following acetylation of the phenolic hydroxyl groups in lignin, acetylated lignin is formed. This product exhibits a characteristic absorption peak at 280 nm, enabling the quantitative determination of lignin content via changes in absorbance.

The pectin content of the samples was quantitatively determined using a Protopectin Content Assay Kit AKSU011C (Beijing Boxbio Science & Technology Co., Ltd., Beijing, China), according to the manufacturer’s instructions, and three experimental repeats were performed. Protopectin is hydrolyzed into soluble pectin in dilute acid solutions and is subsequently converted to galacturonic acid. Galacturonic acid condenses with carbazole to form a purplish–red compound that shows a characteristic absorption peak at 530 nm, allowing the quantitative determination of protopectin content via changes in absorbance.

### 4.7. Fiber Dimension Measurement

Small matchstick-sized pieces for obtaining fiber dimensions were cut from bark from seedling stems or 2-year-old twigs. These pieces were placed in glass tubes and then macerated at 100 °C in a boiling mixture of hydrogen peroxide and glacial acetic acid (1:1 ratio). The pieces were washed with ultrapure water until there was no acid left on them. The fiber length, the fiber diameter, and the lumen diameter were measured after they were stained with 1% O-safranin solution [53]. The dimension of the fibers was measured with a light microscope (Leica, DM6B, Wetzlar, Germany) connected to an image analyzing system (LAS Image Analyzing System SuiteX 3.4.2.18368), and 60 undamaged fibers in each sample were measured.

### 4.8. Tensile Property Test

Bark was cut into dog-bone-shaped specimens. A minimum of 12 replicates (from 6 plants) were tested for bark specimens from seedlings and twig segments at different ages in rubber trees. Then, their thickness was measured at three different locations in the lamina cross-section, using a digital vernier caliper (GuangLu, 111-102v-10G, Guilin, China). The average thickness, multiplied by the width of the specimen surface, provided the cross-sectional area of the tested specimen.

Tensile tests on the specimens were performed at ambient temperature using the computer-controlled MTS Electromechanical Universal Testing Machine (MTS, CMT5504, Shenzhen, China), equipped with a 50 KN load cell. The bark specimens were fixed at a gauge length of 200 mm between special clamps with face serrations to protect the specimens against slippage. Plastic shims were placed between the clamp and the specimen in order to minimize bark tissue damage from the tightening of the clamps. Specimens were first relaxed by lowering the crosshead by 5 mm, and then they were extended at a rate of 1.5 mm s^−1^ until failure. The tensile strength (MPa) was calculated by dividing the load at failure (N) by the cross-sectional area of the specimen [mm^2^, Formula (9)], strain [mm, as Formula (10)], and Young’s modulus [as Formula (11)] were also calculated [45,46].

σ = F/S(9)

where σ represents tensile strength, F is the load at failure, and S is the cross-sectional area of the specimen.

ε = ΔL/L(10)

where ε is the tensile strain, ΔL is the specimen tensile displacement corresponding to the direction parallel to the grain of the bark, and L is the gauge length.

E = σ/ε(11)

where E represents Young’s modulus.

### 4.9. Visual Observation of Bark Bleeding

During chilling stress, the area of bark bleeding in seedlings and twigs was recorded every day. The dimensions of bark bleeding were ascertained using a digital vernier caliper (GuangLu, 111-102v-10G, Guilin, China). At the end of the treatment, bark-bleeding areas in seedlings and twigs were calculated based on the recorded data.

### 4.10. Data Analysis and Statistics

One-way analysis of variance (ANOVA) was applied to the experimental data (Excel 2021). The Duncan’s multivariate range tests were used as a means of making the mean comparison among the different groups of data at *p* < 0.05. All data are presented as the mean ± standard error. The Spearman correlation between chilling resistance, cell-wall chemical components, fiber dimension, tensile properties, and bark-bleeding properties was performed using Origin 2021. Origin 2021 was also used for visualization. Images were embellished using Adobe Illustrator CS6 (v. 16).

The method of the membership function value was utilized to execute a comprehensive evaluation of the photosynthetic pigment content and photosynthetic activity in the bark. The formulas are as follows [54]:

U(X_i_) = (X_i_ − X_min_) /(X_max_ − X_min_) (12)

(13) $$D_{\mathrm{chl}}=\frac{1}{n}\sum_{i=1}^{n}U(X_{i})$$

where U(X_i_) represents the membership function value of the i-th photosynthetic pigment indicator (chl *a*, chl *b*, Tchl, and caro) of the samples, X_i_ denotes the photosynthetic pigment indicator values, and X_max_ and X_min_ are the maximum and minimum values of the i-th pigment indicator values, respectively. D_chl_ stands for the comprehensive evaluation value of the pigment indicators using the membership function method.

## 5. Conclusions

Collectively, we investigated the trade-offs in bark function—focusing on its mechanical properties, photosynthetic potential, and water storage capacity—to explore its roles in the chilling resistance of rubber trees and the mechanism of bark bleeding caused by chilling stress. By comparing the differences in physiological parameters (photosynthetic pigment content and RWC), chemical components, fiber morphologies, and tensile properties in the bark of *Hevea brasiliensis* at different ages, we uncovered the species-specific variations in bark functions according to the annual changing environment. The LT_50_ values in rubber tree bark under chilling stress elucidated increasing chilling resistance with tree aging. This integrative study of the stem age-mediation of seedling responses to chilling stress provides a comprehensive assessment of their photosynthetic potential, water storage capacity, mechanical supports, and protection function trade-offs. Our findings highlight the rule that bark might demonstrate resource allocation tradeoffs between its various functions during tree aging and changes in environmental selective pressures, thereby rendering the simultaneous maximization of two or more functions unattainable. From this point of view, the chilling-induced bark-bleeding symptom in rubber trees could be evidence of these tradeoffs. Altogether, this study underscores the importance of the coordination of structure and function. This research contributes a critical foundation for bark-bleeding mechanisms under chilling stress.

## Figures and Tables

**Table 1:** Statistical values of fiber properties in *Hevea brasiliensis* bark at different ages.

Fiber Properties	GB	SLB	5YB	21YB
FL (μm)	2722.3660 ± 66.4041 c	2786.0260 ± 110.3086 c	3381.9610 ± 93.4299 b	4014.6650 ± 134.4203 a
FW (μm)	19.9108 ± 0.4474 bc	18.8703 ± 0.41 c	22.1818 ± 0.5015 a	20.8010 ± 0.3888 b
DWT(μm)	18.6548 ± 0.4249 bc	17.5515 ± 0.3385 c	21.0406 ± 0.5013 a	19.6665 ± 0.3842 b
LD (μm)	1.2560 ± 0.0571 a	1.3187 ± 0.1808 a	1.1411 ± 0.0264 a	1.1345 ± 0.0274 a
FL/FW	137.7251 ± 4.6514 c	152.1800 ± 6.4443 bc	156.5164 ± 5.3717 b	197.3573 ± 7.8216 a
DWT/LD	16.7325 ± 0.8078 b	16.5424 ± 0.6522 b	19.1053 ± 0.6690 a	17.9940 ± 0.5807 ab
LD/FW	0.0627 ± 0.0029 ab	0.0665 ± 0.0054 a	0.0530 ± 0.0017 b	0.0554 ± 0.0016 b

The data represent the average of ≥60 replicates. The bars show the standard errors. The different lowercase letters in the same column indicate significant differences (Duncan’s test of one-way ANOVA, *p* < 0.05) among the statistical data. FL, fiber length; FW, fiber width; DWT, double-wall thickness; LD, lumen diameter; FL/FW, fiber length/fiber width; DWT/LD, double-wall thickness/lumen diameter; LD/FW, lumen diameter/fiber width.

**Table 2:** Tensile properties in *Hevea brasiliensis* bark at different ages.

Tensile Properties	GB	SLB	5YB	21YB
BT (mm)	0.6271 ± 0.0258 c	0.8327 ± 0.0243 b	1.0993 ± 0.0239 a	1.0209 ± 0.0566 a
ML (N)	5.8500 ± 0.3084 b	4.6123 ± 0.1744 c	7.2080 ± 0.4582 a	7.0164 ± 0.9192 ab
TS (MPa)	9.6647 ± 0.7067 a	5.6038 ± 0.2386 b	6.6170 ± 0.4965 b	6.9109 ± 0.8401 b
EBP (%)	0.3374 ± 0.0291 a	0.2821 ± 0.0299 a	0.2916 ± 0.0180 a	0.3072 ± 0.0477 a
YM (MPa)	3074.6760 ± 212.9404 a	2677.2310 ± 422.0297 a	2283.2720 ± 108.3105 a	2716.7490 ± 472.8373 a

The data represent the average of successful testing specimens. The bars show the standard errors. The different lowercase letters in the same column indicate significant differences (Duncan’s test of one-way ANOVA, *p* < 0.05) among the statistical data. BT, bark thickness. ML, maximum load. TS, tensile strength. EBP, elongation at the break point. YM, Young’s modulus.

**Table 3:** Logistic equation and cold resistance orders in *Hevea brasiliensis* bark.

Sample	Logistic Equation	LT_50_ (°C)	R^2^	Order of Chilling Resistance
GB	y = 1/(1 + 1.7789 × e^0.6622x^)	−0.8695	0.9433 **	4
SLB	y = 1/(1 + 2.1641 × e^0.5932x^)	−1.3021	0.9863 **	3
5YB	y = 1/(1 + 4.0082 × e^0.7931x^)	−1.7504	0.9364 **	2
21YB	y = 1/(1 + 4.7676 × e^0.7661x^)	−2.0387	0.9341 **	1

** indicates extremely significant at *p* < 0.01.

**Table 4:** Statistical values of fiber properties in stem bark of *Hevea brasiliensis* seedlings at different stem ages under chilling stress.

Fiber Properties	GB		SLB	
	0 d	7 d	0 d	7 d
FL (μm)	2722.3660 ± 66.4041 b	3133.6330 ± 88.4555 a	2786.0260 ± 110.3086 b	3140.588 ± 80.059 a
FW (μm)	19.9108 ± 0.4474 a	18.9018 ± 0.4352 a	18.8703 ± 0.41 a	17.6059 ± 0.3794 b
DWT (μm)	18.6548 ± 0.4249 a	17.1258 ± 0.4673 bc	17.5515 ± 0.3385 ab	16.3246 ± 0.3793 c
LD (μm)	1.2560 ± 0.0571 b	2.1262 ± 0.3068 a	1.3187 ± 0.1808 b	1.2813 ± 0.0321 b
FL/FW	137.7251 ± 4.6514 c	164.9380 ± 4.9622 b	152.1800 ± 6.4443 bc	182.3958 ± 5.7221 a
DWT/LD	16.7325 ± 0.8078 a	12.9035 ± 0.6635 b	16.5424 ± 0.6522a	13.3049 ± 0.5021 b
LD/FW	0.0627 ± 0.0029 b	0.1133 ± 0.0169 a	0.0665 ± 0.0054 b	0.0748 ± 0.0025 b

The data represent the average of ≥60 replicates. The bars show the standard errors. The different lowercase letters in the same column indicate significant differences (Duncan’s test of one-way ANOVA, *p* < 0.05) among the statistical data. FL, fiber length; FW, fiber width; DWT, double-wall thickness; LD, lumen diameter; FL/FW, fiber length/fiber width; DWT/LD, double-wall thickness/lumen diameter; LD/FW, lumen diameter/fiber width.

**Table 5:** Tensile properties in stem bark in *Hevea brasiliensis* seedlings at different stem ages under chilling stress.

Tensile Properties	GB		SLB	
	0 d	7 d	0 d	7 d
BT (mm)	0.6271 ± 0.0258 c	0.5771 ± 0.0306 c	0.8327 ± 0.0243 a	0.7247 ± 0.0367 b
ML (N)	5.8500 ± 0.3084 ab	6.6579 ± 0.6656 a	4.6123 ± 0.1744 c	4.9207 ± 0.2767 bc
TS (MPa)	9.6647 ± 0.7067 b	11.7743 ± 1.2474 a	5.6038 ± 0.2386 c	7.1953 ± 0.652 c
EBP (%)	0.3374 ± 0.0291 b	0.5872 ± 0.1417 a	0.2821 ± 0.0299 b	0.2827 ± 0.0377 b
YM (MPa)	3074.6760 ± 212.9404 a	3354.7546 ± 913.5118 a	2677.2310 ± 422.0297 a	3469.5580 ± 579.3064 a

The data represent the average of the successful testing specimens. The bars show the standard errors. The different lowercase letters in the same column indicate significant differences (Duncan’s test of one-way ANOVA, *p* < 0.05) among the statistical data. BT, bark thickness. ML, maximum load. TS, tensile strength. EBP, elongation at the break point. YM, Young’s modulus.

**Table 6:** Data statistics on bark bleeding in *Hevea brasiliensis* bark.

The Situation of Bark Bleeding	GB	SLB	5YB	21YB
NBPs	9	0	85	82
AB (mm^2^)	23.1130	0	789.6621	656.5682
PL	Petiole	/	Bundle scar, lenticel	Bundle scar, lenticel
RBSs/RBTSs	16.6670%	0	100%	100%

NBPs, the number of bark-bleeding positions; AB, the area of bark bleeding; PL, the precise location; RBSs/RBTSs, the rate of bark-bleeding seedlings or 2-year-old twig segments.

## Data Availability

The data that support the results of this study are available on reasonable request from the corresponding authors.

## Supplementary Materials

The following supporting information can be downloaded at: https://www.mdpi.com/article/10.3390/plants14162531/s1, Table S1: Chlorophyll content in *Hevea brasiliensis* bark at different ages; Table S2: Comprehensive evaluation values of photosynthetic pigment content values in bark at different ages; Table S3: RWC values in *Hevea brasiliensis* bark at different ages; Table S4: Cell-wall component content in *Hevea brasiliensis* bark at different ages; Table S5: Different REC values in *Hevea brasiliensis* bark at different ages; Table S6: Chlorophyll content in stem bark of *Hevea brasilensis* seedlings at different stem ages under chilling stress; Table S7: RWC values in stem bark of *Hevea brasilensis* seedlings at different ages under chilling stress; Table S8: Cell-wall component content in stem bark of *Hevea brasilensis* seedlings at different stem ages under chilling stress.
